# Causal effect of gut microbiota on Gastroduodenal ulcer: a two-sample Mendelian randomization study

**DOI:** 10.3389/fcimb.2023.1322537

**Published:** 2023-12-08

**Authors:** Jing Zhang, Yingqiu Hu, Lidong Wu, Qi Zeng, Bin Hu, Zhiqiang Luo, Yibing Wang

**Affiliations:** ^1^ Department of Emergency, The Second Affiliated Hospital, Jiangxi Medical College, Nanchang University, Jiangxi Province, China; ^2^ Queen Mary University of London, Nanchang University, Jiangxi Province, China

**Keywords:** Mendelian randomization, gut microbiota, genome-wide association study, gastroduodenal ulcer, causal relationship

## Abstract

**Background:**

Gastroduodenal ulcers are associated with *Helicobacter pylori* infection and the use of nonsteroidal anti-inflammatory drugs (NSAIDs). However, the causal relationship between gastroduodenal ulcers and gut microbiota, especially specific gut microbiota, remains unclear.

**Methods:**

We conducted an analysis of published data on the gut microbiota and Gastroduodenal ulcer using genome-wide association studies (GWAS). Two-sample Mendelian randomization (MR) analysis was performed to determine the causal relationship between gut microbiota and Gastroduodenal ulcer. Sensitivity, heterogeneity, and pleiotropy analyses were conducted to confirm the accuracy of the research findings.

**Results:**

Our study showed that the abundance of *Enterobacteriaceae*, *Butyricicoccus*, *Candidatus Soleaferrea*, *Lachnospiraceae NC2004 group*, *Peptococcus*, and *Enterobacteriales* was negatively correlated with the risk of Gastroduodenal ulcer. Conversely, the abundance of *Streptococcaceae*, *Lachnospiraceae UCG010*, *Marvinbryantia*, *Roseburia*, *Streptococcus*, *Mollicutes RF9*, and *NB1n* was positively correlated with the risk of Gastroduodenal ulcer. MR analysis revealed causal relationships between 13 bacterial genera and Gastroduodenal ulcer.

**Conclusion:**

This study represents a groundbreaking endeavor by furnishing preliminary evidence regarding the potentially advantageous or detrimental causal link between the gut microbiota and Gastroduodenal ulcer, employing Mendelian Randomization (MR) analysis for the first time. These discoveries have the potential to yield fresh perspectives on the prevention and therapeutic approaches concerning Gastroduodenal ulcer, with a specific focus on the modulation of the gut microbiota.

## 1 Introduction

Gastroduodenal ulcer, also known as peptic ulcers, are characterized by the formation of ulcers within the mucosal lining of the stomach or duodenum. Clinical manifestations typically encompass upper abdominal discomfort, accompanied by symptoms such as nausea, vomiting, and dyspepsia (Malfertheiner et al., 2009). Frequent etiological factors encompass *Helicobacter pylori* infection, the use of nonsteroidal anti-inflammatory drugs (NSAIDs), aspirin, and genetic predisposition (Huang et al., 2002; Graham, 2014; Miftahussurur and Yamaoka, 2015). Notwithstanding our deepening comprehension of the pathophysiological underpinnings of Gastroduodenal ulcer and the progress in therapeutic modalities, the persistent issues of extended treatment duration, post-treatment monitoring, and the specter of recurrence continue to pose substantial challenges. These challenges not only result in financial burdens on patients and their families but also exert a profound impact on patients’ quality of life and occupational productivity (Lanas and Chan, 2017). Furthermore, Gastroduodenal ulcer can give rise to complications, notably perforations, with potentially grave consequences for patients’ well-being (de Boer, 1997). Hence, there is an urgent demand for the exploration of additional therapeutic modalities for Gastroduodenal ulcer with the aim of ameliorating the socioeconomic strain on both families and society.

The gut microbiota constitutes an intricate and diversified microbial consortium inhabiting the human gastrointestinal tract. It encompasses a wide array of microorganisms, encompassing bacteria, fungi, viruses, and parasites. Notably, bacteria represent the predominant constituents among them (Sender et al., 2016). The gut microbiota assumes a pivotal role in the human organism. It is involved in the synthesis and secretion of diverse bioactive substances, including vitamins, enzymes, and antibiotics, that confer numerous advantages to human physiology. Furthermore, the gut microbiota actively participates in the processes of digestion, the maintenance of intestinal health, and the preservation of microbial diversity and equilibrium within the gut. These functions are of paramount importance for overall health (Thursby and Juge, 2017). Furthermore, the gut microbiota exerts a pivotal influence on the maturation and modulation of the human immune system, thereby actively contributing to the preservation of immune homeostasis (Belkaid and Hand, 2014). The correlation between the gut microbiota and Gastroduodenal ulcer is an ongoing subject of research. Studies have indicated disparities in the gut microbiota composition between Gastroduodenal ulcer patients and healthy individuals (Chen X. et al., 2018). For instance, patients with Gastroduodenal ulcer may exhibit an overabundance of pathogenic bacteria, such as Helicobacter pylori, in their gut microbiota. Moreover, the disturbance of gut microbiota equilibrium may be associated with Gastroduodenal ulcer development (Schulz et al., 2018).

Due to the potential influence of uncontrolled confounding variables, traditional research methodologies often face limitations in elucidating the precise association between gut microbiota and Gastroduodenal ulcer. Consequently, the establishment of a causal link between gut microbiota and Gastroduodenal ulcer remains inconclusively characterized. Mendelian randomization (MR) serves as a viable approach to assess the presence of a causal relationship between the exposure and the outcome. This is achieved by employing single-nucleotide polymorphisms (SNPs) closely linked to relevant risk factors as instrumental variables (IVs) (Emdin et al., 2017). The reliability of the causal relationship within this methodology stems from the fact that the random allocation of alleles during embryonic meiosis remains largely impervious to the influence of most confounding variables (Zhang et al., 2023). To address the current void in Mendelian randomization (MR) analyses concerning the causal nexus between gut microbiota and Gastroduodenal ulcer, we undertook a comprehensive genome-wide association study (GWAS) followed by a two-sample Mendelian randomization (MR) investigation. This research endeavors to furnish a more profound comprehension of the influence exerted by gut microbiota on Gastroduodenal ulcer and to proffer robust scientific substantiation conducive to the prevention and management of Gastroduodenal ulcer through gut microbiota modulation.

## 2 Methods

### 2.1 Study design and data sources

**Figure 1:**
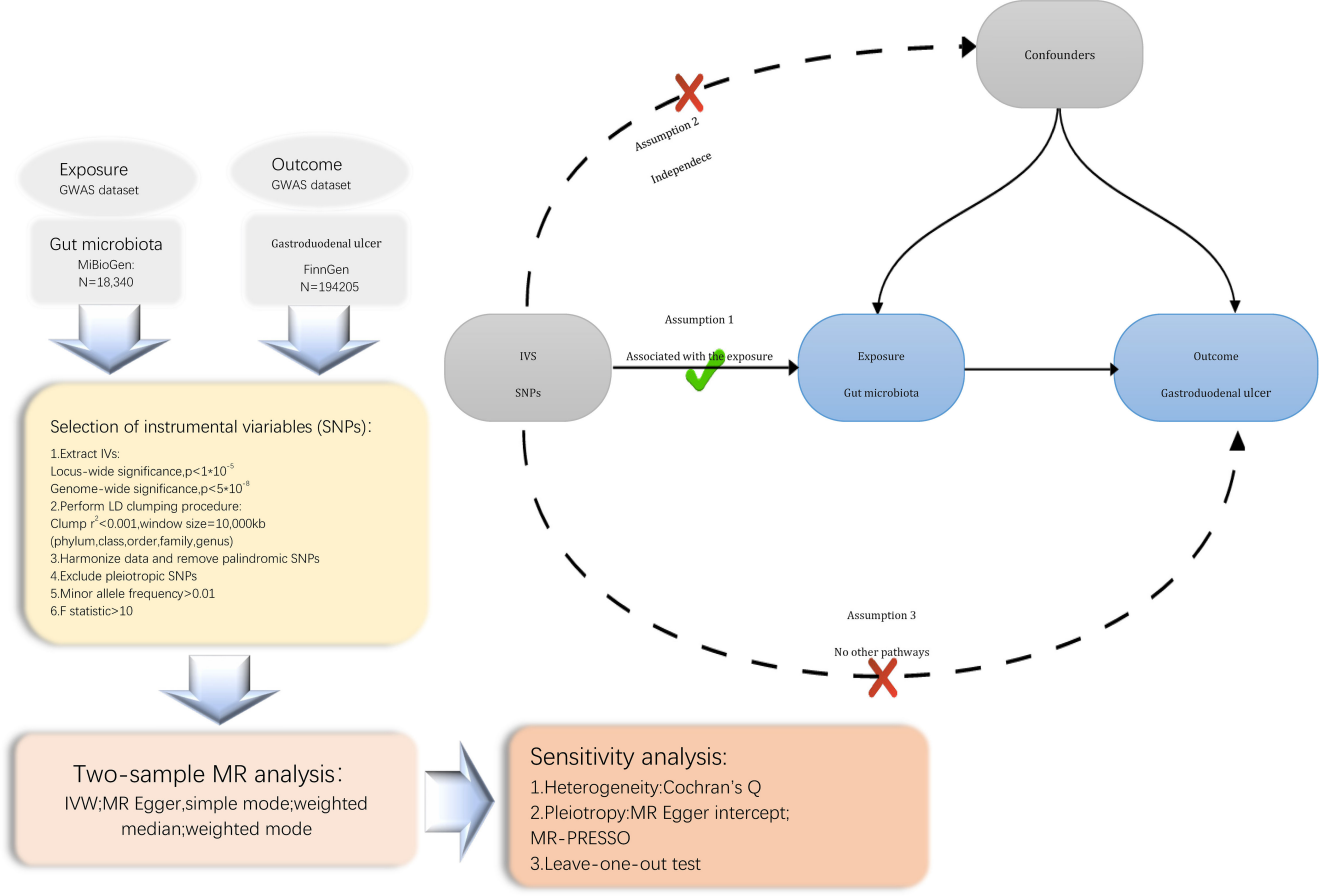
Flowchart of the present MR study and major assumptions. MR, Mendelian randomization; GWAS, genome-wide association study; SNPs, single nucleotide polymorphisms; IVW, inverse-variance weighted; LD, linkage disequilibrium; MR-PRESSO, MR pleiotropy residual sum and outlier.

We conducted a Mendelian randomization (MR) study to explore the causal relationship between the gut microbiota and Gastroduodenal ulcer. The schematic diagram of our study process is shown in **Figure 1**. In summary, we extracted data from summary statistics of genome-wide association studies (GWAS) to identify genetic variations associated with the exposure, which were subsequently used as instrumental variables (IVs). We performed a sequential two-sample MR analysis employing five distinct MR methodologies. Finally, a comprehensive set of sensitivity analysis metrics, including tests for heterogeneity, pleiotropy, and leave-one-out analysis, were applied to assess significant associations.

Summary-level genomic data of the gut microbiota were acquired from the MiBioGen study (data from https://mibiogen.gcc.rug.nl/) (Kurilshikov et al., 2021).This study represented the largest and most diverse genome-wide meta-analysis of the gut microbiota to date, encompassing genome-wide genotyping data and 16S fecal microbiota profiles from 24 cohorts, comprising a total of 18,340 individuals. The majority of participants in the study were of European descent (N=13,266). Profiling of microbial composition was achieved through targeted sequencing of the V4, V3-V4, and V1-V2 regions of the 16S rRNA gene. Subsequently, taxonomic classification was performed utilizing direct taxonomic binning. Following the processing of 16S microbiome data, a total of 211 taxa were identified, encompassing 131 genera, 35 families, 20 orders, 16 classes, and 9 phyla. Comprehensive information regarding the microbiota dataset can be found in the original investigation (Kurilshikov et al., 2021).

The GWAS summary data on Gastroduodenal ulcer (finn-b-K11_GASTRODUOULC) were obtained from the FinGen, including 9216 cases of Gastroduodenal ulcer and 320387 controls (Kurki et al., 2023). To enhance the reliability of our findings, we carried out an extensive search in the “ieu open gwas project” focusing on data related to Gastroduodenal ulcer. After careful screening, we selected the dataset with the largest sample size, which not only had a large sample size but also contained detailed information about Gastroduodenal ulcer. Using this large sample dataset, we can analyze and study the relevant features and risk factors of Gastroduodenal ulcer more accurately. Thus, our results will be more persuasive and will provide stronger evidence for research and practices in relevant fields (data from https://gwas.mrcieu.ac.uk/).

### 2.2 Instrumental variable selection

To ensure the accuracy and validity of our conclusions regarding the causal relationship between gut microbiota and Gastroduodenal ulcer, we implemented a series of quality control procedures to filter instrumental variables (IVs). Firstly, we selected single-nucleotide polymorphisms (SNPs) with significant associations to the gut microbiome as IVs. SNPs were chosen based on two distinct thresholds. In order to obtain a comprehensive overview and enhance the explained phenotypic variability, we included a set of SNPs with locus-wide significance levels below 1×10-5 as IVs. Additionally, for secondary analysis, another set of SNPs with genome-wide significance (p<5×10-8) were selected as IVs, but we did not find enough sample size in our experiments. Secondly, to ensure the independence of the selected IVs and minimize the impact of linkage disequilibrium that violates the random allele assignment, we configured the clumping procedure with parameters set to r2<0.001 and kb=10,000kb. Thirdly, If exposure-related SNPs were not identified in the outcome genome-wide association study (GWAS) results, proxy SNPs highly correlated with the target variant (r2>0.8) were identified through the SNiPA website (Arnold et al., 2015). However, it’s important to note that such a scenario did not occur in our analysis. Fourthly, SNPs with palindromic properties and incompatible alleles were disqualified from the Mendelian Randomization (MR) analysis. Fifthly, in order to satisfy the second key assumption of MR (independence from confounders), we conducted a manual inspection and exclusion of SNPs significantly associated (p<5×105) with potential confounding factors using the PhenoScanner GWAS database (Staley et al., 2016; Kamat et al., 2019). SNPs such as rs166849 and rs6494306 were eliminated because they were associated with past smoking and type 2 diabetes. In addition, SNPs rs2952251 were associated with anxiety, past smoking, and mood swings, SNPs rs62532512 were associated with past smoking, mood swings, and misery, and SNP rs17708276 were associated with worry, tension, and misery, and all SNPs rs2952251, rs62532512, and rs17708276 were also deleted. Sixthly, a minimum minor allele frequency threshold of 0.01 was enforced. Lastly, to mitigate weak instrumental bias, the F-statistic was computed for each SNP, and any SNPs with F-statistics below 10 were discarded (Burgess and Thompson, 2011). The F-statistic is expressed as R2 (n-k-1)/k (1-R2), with n representing the sample size, k denoting the number of IVs, and R2 signifying the variance explained by the IVs.

### 2.3 Effect size estimate

We conducted a two-sample Mendelian randomization (MR) analysis to explore the causal relationship between gut microbiome features and the risk of Gastroduodenal ulcer.When multiple IVs were involved in a gut microbiota feature, we adopted the inverse-variance weighted (IVW) test as the primary analytical approach, complemented by other methodologies, including MR-Egger, simple mode, weighted median, and weighted mode (Burgess et al., 2013). To comprehensively assess the influence of the gut microbiome on Gastroduodenal ulcer, the meta-analysis technique known as IVW converted the outcome effects of IVs on exposure effects into a weighted regression model with an intercept constrained to zero. In the absence of horizontal pleiotropy, IVW yielded unbiased estimates by mitigating the influence of confounding variables (Holmes et al., 2017). It is noteworthy that the MR-Egger method may be susceptible to the influence of outlier genetic variables, potentially leading to incorrect estimations. However, even when all selected IVs are invalid, the MR-Egger approach can still produce unbiased estimates (Bowden et al., 2016b). The simple mode offers robustness against pleiotropy effects, although it may be less statistically powerful than IVW (Milne et al., 2017). The weighted median method, when at least 50% of data from valid instruments are available, is capable of providing precise and reliable effect estimates (Bowden et al., 2016a). In situations involving genetic variables that violate the pleiotropy assumption, the weighted mode method can be adapted (Hartwig et al., 2017).

### 2.4 Sensitivity analysis

To assess the potential impact of heterogeneity and pleiotropy among instrumental variables (IVs) on MR results, a comprehensive set of sensitivity analyses was undertaken to ascertain the robustness of our significant findings. Heterogeneity among the selected genetic instruments was quantified using Cochran’s Q test and visualized through funnel plots. Furthermore, we probed for potential horizontal pleiotropic effects of the included IVs, employing both the MR Egger intercept and the Mendelian randomization pleiotropy residual sum and outlier (MR-PRESSO) global test. Concurrently, we performed a leave-one-out sensitivity analysis to validate the precision and robustness of causal effect estimates, ensuring that our MR estimates were not unduly influenced by highly influential SNPs. In addition, the MR Steiger directionality test was employed to infer the causal direction (Hemani et al., 2017). Credible causal links were identified when the variance explained by the IVs on the exposure exceeded that on the outcome. All statistical analyses in our investigation, encompassing both MR and sensitivity analyses, were executed using the R packages “TwoSampleMR” and “MRPRESSO” within the publicly available R software (version 4.3.1).

## 3 Results

### 3.1 Instrumental variable selection

In our study, we commenced by choosing 211 bacterial taxa as the subjects of investigation. To guarantee the adherence of instrumental variables (IVs) to the established criteria, we conducted a rigorous screening process to eliminate instrumental variables that exhibited significant associations with the study objectives. In order to satisfy the second critical assumption of Mendelian Randomization (MR), which pertains to the independence of confounding factors, we further utilized the PhenoScanner GWAS database for a meticulous manual examination. This allowed us to identify and subsequently exclude instrumental variables that displayed significant associations with potential confounding factors. During this process, we carefully identified instrumental variables significantly linked to confounding factors and duly removed these variables to ensure the precision of our research outcomes. These procedures are essential in ensuring the independence of the instrumental variables we employed, allowing us to effectively infer causal relationships. Subsequently, the remaining data underwent re-analysis utilizing the aforementioned methodologies. The results of the analysis indicated that when employing the Inverse Variance Weighting (IVW) method as the primary analytical approach, the p-value associated with *Rikenellaceae* exceeded 0.05. As a result, *Rikenellaceae* and all the included Single Nucleotide Polymorphisms (SNPs) were removed. During the final screening phase, we rigorously selected 156 Single Nucleotide Polymorphisms (SNPs) as instrumental variables (see **Supplementary Table S1**). These instrumental variables underwent meticulous filtration to guarantee their effectiveness and reliability within the context of our study. It is noteworthy that all instrumental variables exhibited F-values exceeding 10, signifying their robust predictive capacity in explaining variables. Importantly, this observation underscores that our instrumental variables are not weak, and they can be effectively employed to address endogeneity issues (see **Table 1**). These outcomes bolster our confidence in the validity of our research findings and furnish robust support for subsequent analyses.

**Table 1:** MR estimates for the association between gut microbiota and Gastroduodenal ulcer (*p *< 1 × 10^−5^).

Level	Microbiota	SNPs	Methods	Bate	OR(95%CI)	p value
**family**	Enterobacteriaceae	11	MR Egger	-0.26	0.77(0.22,2.71)	0.696
			Weighted median	-0.35	0.71(0.50,0.99)	0.043
			Inverse variance weighted	-0.29	0.75(0.58,0.97)	0.031
			Simple mode	-0.42	0.66(0.40,1.09)	0.136
			Weighted mode	-0.40	0.67(0.43,1.05)	0.114
**family**	Streptococcaceae	17	MR Egger	0.13	1.14(0.55,2.35)	0.724
			Weighted median	0.2	1.22(0.93,1.60)	0.153
			Inverse variance weighted	0.29	1.34(1.09,1.63)	0.004
			Simple mode	0.09	1.10(0.66,1.82)	0.721
			Weighted mode	0.13	1.14(0.74,1.75)	0.566
**genus**	Butyricicoccus	9	MR Egger	-0.38	0.68(0.39,1.19)	0.223
			Weighted median	-0.10	0.91(0.63,1.31)	0.602
			Inverse variance weighted	-0.30	0.74(0.57,0.96)	0.024
			Simple mode	0.04	1.04(0.54,2.01)	0.915
			Weighted mode	0.05	1.05(0.50,2.19)	0.906
**genus**	CandidatusSoleaferrea	16	MR Egger	-0.17	0.84(0.46,1.54)	0.587
			Weighted median	-0.11	0.89(0.75,1.07)	0.215
			Inverse variance weighted	-0.13	0.88(0.77,1.00)	0.045
			Simple mode	-0.14	0.87(0.66,1.16)	0.367
			Weighted mode	-0.16	0.85(0.63,1.16)	0.331
**genus**	LachnospiraceaeNC2004group	10	MR Egger	-0.12	0.89(0.45,1.75)	0.735
			Weighted median	-0.25	0.78(0.62,0.97)	0.027
			Inverse variance weighted	-0.21	0.81(0.69,0.95)	0.012
			Simple mode	-0.32	0.72(0.51,1.02)	0.102
			Weighted mode	-0.32	0.73(0.50,1.07)	0.141
**genus**	LachnospiraceaeUCG010	12	MR Egger	-0.25	0.78(0.39,1.56)	0.495
			Weighted median	0.36	1.43(1.07,1.90)	0.015
			Inverse variance weighted	0.29	1.33(1.07,1.66)	0.011
			Simple mode	0.38	1.47(0.89,2.42)	0.162
			Weighted mode	0.38	1.47(0.90,2.40)	0.155
**genus**	Marvinbryantia	11	MR Egger	0.18	1.19(0.52,2.76)	0.688
			Weighted median	0.20	1.22(0.91,1.64)	0.193
			Inverse variance weighted	0.24	1.27(1.01,1.58)	0.037
			Simple mode	0.20	1.22(0.75,1.97)	0.439
			Weighted mode	0.20	1.22(0.77,1.95)	0.415
**genus**	Peptococcus	16	MR Egger	-0.32	0.73(0.46,1.16)	0.204
			Weighted median	-0.10	0.91(0.77,1.07)	0.244
			Inverse variance weighted	-0.15	0.86(0.76,0.97)	0.018
			Simple mode	0.01	1.01(0.75,1.35)	0.971
			Weighted mode	0.03	1.03(0.78,1.35)	0.861
**genus**	Roseburia	17	MR Egger	0.69	1.99(1.09,3.62)	0.040
			Weighted median	0.17	1.18(0.86,1.62)	0.295
			Inverse variance weighted	0.26	1.29(1.04,1.61)	0.021
			Simple mode	0.01	1.01(0.55,1.86)	0.978
			Weighted mode	-0.01	0.99(0.58,1.69)	0.976
**genus**	Streptococcus	16	MR Egger	0.11	1.12(0.51,2.45)	0.784
			Weighted median	0.17	1.18(0.88,1.58)	0.268
			Inverse variance weighted	0.24	1.28(1.03,1.57)	0.023
			Simple mode	0.03	1.03(0.63,1.70)	0.909
			Weighted mode	0.09	1.09(0.70,1.70)	0.711
**order**	Enterobacteriales	11	MR Egger	-0.26	0.77(0.22,2.71)	0.696
			Weighted median	-0.35	0.71(0.50,0.99)	0.042
			Inverse variance weighted	-0.29	0.75(0.58,0.97)	0.031
			Simple mode	-0.42	0.66(0.40,1.07)	0.125
			Weighted mode	-0.40	0.67(0.43,1.06)	0.117
**order**	MollicutesRF9	16	MR Egger	0.16	1.17(0.70,1.97)	0.554
			Weighted median	0.18	1.19(0.95,1.49)	0.127
			Inverse variance weighted	0.23	1.26(1.07,1.48)	0.006
			Simple mode	0.13	1.14(0.75,1.73)	0.543
			Weighted mode	0.13	1.14(0.80,1.64)	0.484
**order**	NB1n	15	MR Egger	0.03	1.03(0.60,1.76)	0.926
			Weighted median	0.21	1.23(1.05,1.45)	0.011
			Inverse variance weighted	0.19	1.21(1.07,1.36)	0.002
			Simple mode	0.31	1.37(1.03,1.81)	0.047
			Weighted mode	0.31	1.36(1.02,1.81)	0.051

### 3.2 Causal impact of gut microbiota on gastroduodenal ulcer

Based on our research findings, we have identified causal relationships between 13 bacterial genera and the risk of Gastroduodenal ulcer. Notably, several bacterial taxa with high predicted abundance exhibited significant correlations with the risk of Gastroduodenal ulcer.

**Figure 2:**
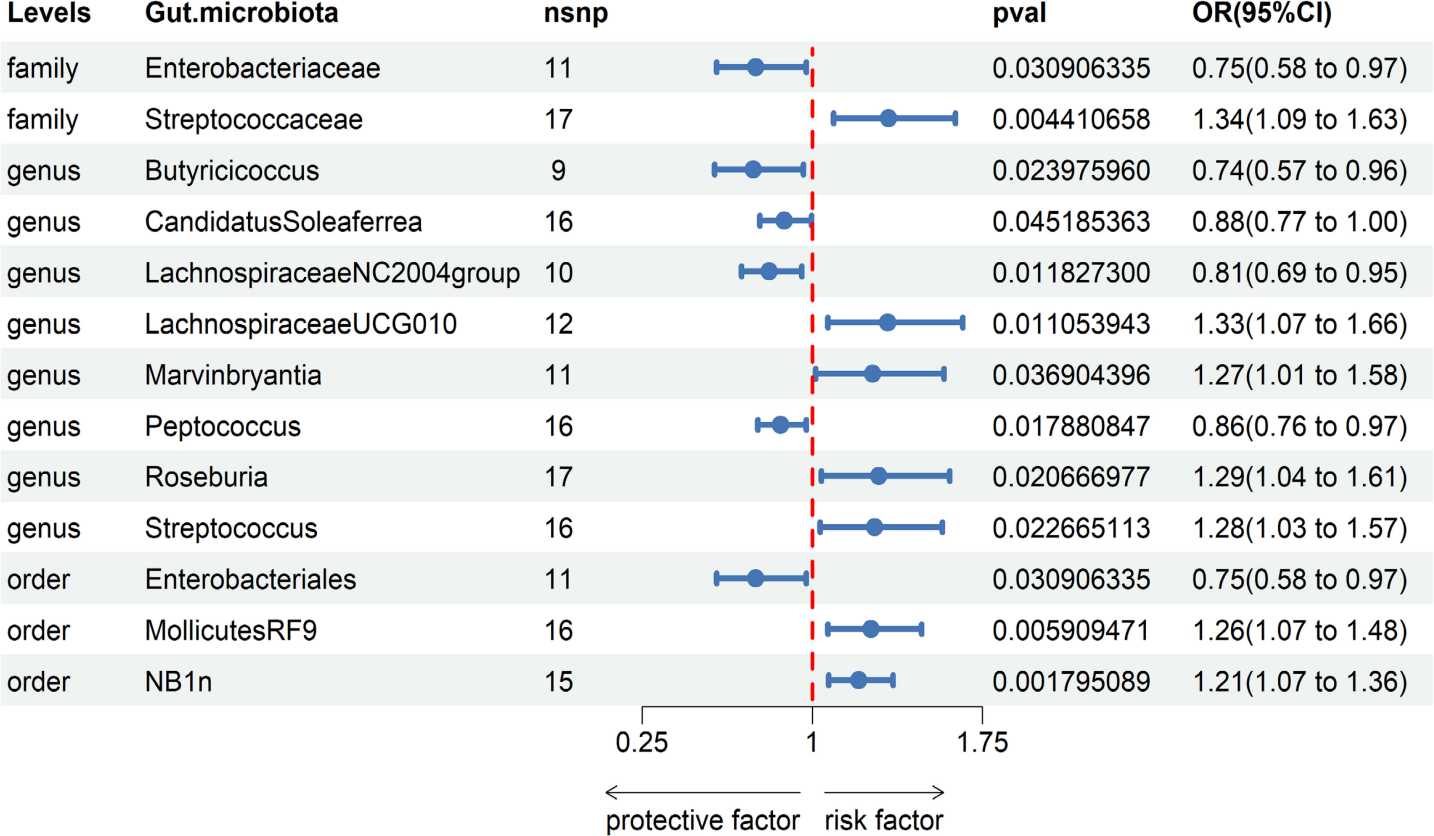
Associations of genetically predicted Gastroduodenal ulcer with sepsis risk using IVW method SNPs, single nucleotide polymorphisms; OR, odds ratio; CI, confidence interval.

Specifically, a higher abundance of *Enterobacteriaceae* (OR: 0.75, 95% CI: 0.58-0.97, p=0.031)was associated with a reduced risk of Gastroduodenal ulcer. Similarly, increased abundances of *Butyricicoccus* (OR: 0.74, 95% CI: 0.57-0.96, p=0.024), *Candidatus Soleaferrea* (OR: 0.88, 95% CI: 0.77-1.00, p=0.045), *Lachnospiraceae NC2004 group* (OR: 0.81, 95% CI: 0.69-0.95, p=0.012), *Peptococcus* (OR: 0.86, 95% CI: 0.76-0.97, p=0.018), and *Enterobacteriales* (OR: 0.75, 95% CI: 0.58-0.97, p=0.031) were associated with a decreased risk of Gastroduodenal ulcer (see **Figure 2**, **Table 1**).

**Table 2:** Evaluation of heterogeneity and directional pleiotropy using different methods.

Level	Microbiota	Heterogeneity	Horizontal pleiotropy	
		Cochran’s Q p	MR-Egger intercept p	MR-PRESSO global test p
**family**	Enterobacteriaceae	0.313	0.237	0.357
	Streptococcaceae	0.785	0.739	0.801
**genus**	Butyricicoccus	0.401	0.314	0.351
	CandidatusSoleaferrea	0.782	0.720	0.792
	LachnospiraceaeNC2004group	0.744	0.660	0.737
	LachnospiraceaeUCG010	0.900	0.982	0.899
	Marvinbryantia	0.783	0.705	0.793
	Peptococcus	0.264	0.239	0.270
	Roseburia	0.359	0.441	0.362
	Streptococcus	0.559	0.492	0.592
**order**	Enterobacteriales	0.313	0.237	0.357
	MollicutesRF9	0.771	0.713	0.786
	NB1n	0.659	0.612	0.678

In contrast, higher abundances of *Streptococcaceae* (OR: 1.34, 95% CI: 1.09-1.83, p=0.004), *Lachnospiraceae UCG010* (OR: 1.33, 95% CI: 1.07-1.66, p=0.011), *Marvinbryantia* (OR: 1.27, 95% CI: 1.01-1.58, p=0.037), *Roseburia* (OR: 1.29, 95% CI: 1.04-1.61, p=0.021), *Streptococcus* (OR: 1.28, 95% CI: 1.03-1.57, p=0.023), *Mollicutes RF9* (OR: 1.26, 95% CI: 1.07-1.48, p=0.006), and *NB1n* (OR: 1.21, 95% CI: 1.07-1.36, p=0.002) were associated with an elevated risk of Gastroduodenal ulcer. These findings suggest that increased abundances of these gut microbiota may be linked to an increased risk of Gastroduodenal ulcer (see **Figure 2**, **Table 1**).

### 3.3 Sensitivity analysis

Our analysis of the relationship between gut microbiota and Gastroduodenal ulcer has revealed a total of 13 causal associations. Initially, we employed Cochran’s Q test to assess the heterogeneity of all instrumental variables (IVs). The results indicated no significant heterogeneity (P>0.05), signifying consistent effects of the selected instrumental variables (IVs) across various studies (refer to **Table 2**).

To further validate the instrumental variables (IVs), we performed MR-Egger intercept tests and MR-PRESSO tests. Encouragingly, all p-values exceeded 0.05, indicating the absence of horizontal pleiotropy and the detection of outliers by MR-PRESSO (refer to **Table 2**). This absence of outliers further bolsters our confidence in the instrumental variables (IVs). These sensitivity analyses confirm that the selected instrumental variables (IVs) exhibit good heterogeneity and lack horizontal pleiotropy, effectively assisting in addressing endogeneity concerns.

The scatter plot illustrates the relationship between distinct gut microbiota and the incidence of Gastroduodenal ulcer. *Enterobacteriaceae*, *Butyricicoccus*, *Candidatus Soleaferrea*, *Lachnospiraceae NC2004 group*, *Peptococcus*, and *Enterobacteriales* are considered to have a protective effect, indicating a negative correlation with the occurrence of Gastroduodenal ulcer. Conversely, *Streptococcaceae*, *Lachnospiraceae UCG010*, *Marvinbryantia*, *Roseburia*, *Streptococcus*, *Mollicutes RF9*, and *NB1n* are associated with a non-protective effect, demonstrating a positive correlation with Gastroduodenal ulcer incidence.

The scatter plot also displays the weights obtained through various MR analysis methods (IVW, MR-Egger, weighted median, weighted mode, and simple mode). These lines represent the non-protective or protective relationships between diverse gut microbiota and Gastroduodenal ulcer. An upward trend from left to right suggests a non-protective relationship with Gastroduodenal ulcer, while a downward trend indicates a protective relationship with Gastroduodenal ulcer (**Supplementary Figures S3**).

Through leave-one-out analysis, we identified no potential outliers among all instrumental variables (IVs), signifying that the established causal relationships remain unaffected by individual instrumental variables (IVs) (**Supplementary Figures S1**, **S2**). This finding further bolsters the reliability of the association between gut microbiota and Gastroduodenal ulcer.

## 4 Discussion

To the best of our knowledge, this study represents the inaugural exploration into the causal association between gut microbiota and Gastroduodenal ulcer, utilizing publicly available GWAS data. Employing two-sample MR methods, we have effectively uncovered 13 causal relationships connecting gut microbiota to Gastroduodenal ulcer risk. This discovery furnishes pivotal scientific substantiation, advancing our comprehension of the impact of gut microbiota on Gastroduodenal ulcer etiology. These findings underscore the integral role played by the gut microbiota composition in Gastroduodenal ulcer. Further investigations hold the potential to deepen our insights into how these microbial entities influence the onset and progression of Gastroduodenal ulcer. Moreover, these revelations proffer novel perspectives for Gastroduodenal ulcer prevention and treatment strategies, including the prospect of modulating gut microbiota composition to enhance gastrointestinal well-being and curtail Gastroduodenal ulcer risk.

In our study, a series of analyses have indicated that a high abundance of *Enterobacteriaceae* may confer protection against Gastroduodenal ulcer. Previous research has demonstrated that *Enterobacteriaceae* exhibits resilience in acidic environments by inducing a low pH-triggered lysine decarboxylase system (CadB-CadA system). This mechanism converts lysine into cadaverine, an alkaline amine, which is subsequently released from the cells via CadB, leading to a reduction in extracellular hydrogen ion concentration (McGowan et al., 1996). The reduction in hydrogen ion concentration plays a significant role in effectively controlling the occurrence of Gastroduodenal ulcer. This discovery enhances our understanding of the protective capacity of *Enterobacteriaceae* against Gastroduodenal ulcer. Further investigation into the mechanisms underlying the protective role of *Enterobacteriaceae* in Gastroduodenal ulcer can pave the way for the development of more effective treatments. For instance, by intervening in the CadB-CadA system, we can potentially augment the survival capabilities of *Enterobacteriaceae*, thereby strengthening its protective effects against Gastroduodenal ulcer. Such interventions could have implications for the prevention and treatment of these ulcerative conditions. It’s worth noting that the gut’s indigenous microbial population includes *Proteobacteria*, a major constituent of the gut microbiota (Kim et al., 2017). In a study investigating alterations in the gut microbial community following a 14-day bismuth quadruple therapy for peptic ulcers, substantial changes were observed in the gut microbiota at the phylum level after the treatment period. There was a notable reduction in the abundance of specific gut bacteria at the phylum level. However, it is noteworthy that the abundance of *Proteobacteria*, which includes *Enterobacteriaceae*, exhibited a relative increase after the treatment (Zhou et al., 2020). The observed increase in *Proteobacteria* abundance implies a potential protective effect against Gastroduodenal ulcer. Notably, our study revealed that *Enterobacterales*, a taxonomic order within the *Proteobacteria* phylum, is associated with this increase, suggesting that a higher abundance of *Enterobacterales* may correspond to a reduced incidence of Gastroduodenal ulcer. Furthermore, it is essential to underline that *Enterobacteriaceae*, which is a family within *Proteobacteria*, aligns with these findings, further corroborating our research results.

In a study evaluating the efficacy of *Helicobacter pylori* eradication therapy for Gastroduodenal ulcer induced by this bacterium, researchers focused on a group of patients diagnosed with *Helicobacter pylori* infection who had not undergone any prior treatment. Within this cohort, *Butyricicoccus* was detected in the gut microbiota. Subsequent to treatment, a notable reduction in the abundance of *Butyricicoccus* was observed in comparison to both the uninfected *Helicobacter pylori* group and the control group devoid of severe digestive system ailments. Interestingly, the study identified a substantial increase in the abundance of *Butyricicoccus* among patients with Gastroduodenal ulcer before treatment in contrast to after treatment (Cui et al., 2022). This implies that an elevated abundance of *Butyricicoccus* may play a role in the development of Gastroduodenal ulcer, which contrasts with our findings. To reconcile this inconsistency, a more comprehensive understanding of the underlying mechanisms and principles is required to better elucidate the impact of *Butyricicoccus* on Gastroduodenal ulcer.

Xia Chen et al. conducted a study indicating a notably higher abundance of *Streptococcus* in patients with gastroduodenal ulcer. This observation implies that an elevated abundance of *Streptococcus* might be a risk factor for gastroduodenal ulcer, aligning with our own findings (Chen X. et al., 2018). It’s important to highlight that prior studies have detected *Mollicutes* in patients with chronic gastritis, but its abundance is comparatively lower in healthy individuals (Nascimento et al., 2021). This indicates that *Mollicutes* may have an impact on the occurrence of Gastroduodenal ulcer. Both *RF9* and *NB1n* are categorized under *Mollicutes*, and our findings align with the notion that *RF9* and *NB1n* may also exhibit a positive correlation with Gastroduodenal ulcer.

*Candidatus Soleaferrea* belongs to the *Candida* genus. In an experiment involving rats induced with cysteamine to induce Gastroduodenal ulcer perforation, the group administered with *Candida* exhibited a significantly higher probability of Gastroduodenal ulcer perforation compared to the group administered with normal saline. Furthermore, the area of Gastroduodenal ulcer was also larger in the *Candida*-administered group than in the normal saline group. These findings from the cysteamine-induced Gastroduodenal ulcer experiment indicate that *Candida* can significantly exacerbate Gastroduodenal ulcer (Nakamura et al., 2007). Additionally, there have been studies indicating that *Candida* infection is present in some patients with gastric-duodenal ulcers. Moreover, in cases where patients have both Gastroduodenal ulcer and Barrett’s ulcers, *Candida* is observed exclusively in those with Gastroduodenal ulcer (Kalogeropoulos and Whitehead, 1988). Nonetheless, our experimental results revealing a negative correlation between increased *Candidatus Soleaferrea* abundance and Gastroduodenal ulcer contradict our initial hypothesis. This suggests the presence of other factors or mechanisms that may influence the relationship between *Candidatus Soleaferrea* and Gastroduodenal ulcer. Therefore, further research is warranted to comprehensively comprehend the association between *Candidatus Soleaferrea* and Gastroduodenal ulcer. This may involve investigating other potential microbiota alterations, host genetic factors, environmental influences, and more to elucidate the specific role of *Candidatus Soleaferrea* in Gastroduodenal ulcer occurrence. The findings from these studies will contribute to a deeper understanding of the interaction between *Candidatus Soleaferrea* and Gastroduodenal ulcer, offering fresh insights into potential treatment strategies and preventive measures.

*Streptococcaceae*, *Lachnospiraceae NC2004 group*, *Lachnospiraceae U-CG010*, *Marvinbryantia*, *Peptococcus*, and *Roseburia* all fall within the *Firmicutes* phylum. *Firmicutes* is a prevalent bacterial phylum typically identified in the human gut. The gut microbiota forms a multifaceted ecosystem comprising diverse microorganisms that exert significant influences on human health and disease (Belkaid and Hand, 2014; Sender et al., 2016; Thursby and Juge, 2017). Recent studies have indicated that patients infected with *Helicobacter pylori* tend to exhibit higher *Firmicutes* abundance in their gut microbiota prior to treatment. However, following a 14-day course of bismuth therapy, significant alterations occur within the gut microbial community, marked by a substantial reduction in *Firmicutes* abundance (Chen L. et al., 2018). In a study involving mice with *Helicobacter pylori*-induced gastritis, researchers observed an elevated abundance of *Firmicutes*. These findings indicate a potential positive correlation between increased *Firmicutes* abundance and the risk of Gastroduodenal ulcer (Lofgren et al., 2011). In our study, we identified a positive correlation between the abundance of *Lachnospiraceae UCG010*, *Marvinbryantia*, *Streptococcaceae*, and *Roseburia* and the incidence of Gastroduodenal ulcer. This suggests that an increase in the abundance of these bacteria may be associated with a higher risk of Gastroduodenal ulcer. However, the specific relationship between *Firmicutes* and Gastroduodenal ulcer remains unclear due to limited research in this area. The scientific community has yet to establish a consensus on this matter, necessitating further investigation to confirm these findings.Conversely, our research revealed a negative correlation between the abundance of *Lachnospiraceae NC2004 group*, and *Peptococcus* and Gastroduodenal ulcer. However, due to the scarcity of relevant studies, we do not have a comprehensive understanding of the specific relationship between these bacterial genera and Gastroduodenal ulcer. Thus, additional research is warranted to elucidate the associations between these genera and the occurrence and progression of Gastroduodenal ulcer. Through in-depth investigations and experiments, we can gain a better understanding of how these bacterial genera contribute to the development of Gastroduodenal ulcer.

Our study employed Mendelian Randomization (MR) analysis methods, which, in comparison to traditional observational studies, can mitigate the influence of confounding factors on the outcomes. We conducted an assessment of the causal relationship between gut microbiota and Gastroduodenal ulcer, specifically at the phylum level. This analysis serves as a foundational framework for future investigations into specific microbial strains, thereby contributing to a deeper comprehension of the pathogenesis of Gastroduodenal ulcer. Our study’s findings offer new insights and potential approaches for the future diagnosis and treatment of Gastroduodenal ulcer. Simultaneously, in order to assess the potential impact of heterogeneity and pleiotropy among instrumental variables on the MR results, we conducted an extensive sensitivity analysis, which further bolsters the reliability of our findings.

Our study has certain limitations that should be taken into account. Firstly, the participants included in the GWAS meta-analysis database were predominantly of European descent, with a limited amount of data from other ethnic groups regarding their gut microbiota. This discrepancy may have influenced our research results, as the composition of gut microbiota can vary among different ethnic groups. And, due to the fact that most of the GWAS data comes from individuals of European descent, even if there may be interference from population stratification, the results of this study may not be applicable to other populations of non-European ancestry. Secondly, in our study, 16S rRNA gene sequencing enables resolution only at the phylum level, preventing us from further exploring the causal relationship between gut microbiota and gastric ulcers at the species level. Moreover, concerning sample size, gut microbiota Genome-Wide Association Studies (GWAS) are in an early stage, with relatively few loci associated with gastric ulcers. To conduct sensitivity analysis and test at various significance levels, more genetic variations need inclusion as instrumental variables. Further exploration requires analysis at higher taxonomic levels, such as order, class, and phylum, which might limit the comprehensive study of specific impacts of individual bacterial species. Thirdly, using a limited number of gut microbiota Single Nucleotide Polymorphisms (SNPs) as instrumental variables, there’s a possibility that our study results could be influenced by weak instrument bias, despite all genetic instruments being associated with the exposure (F-statistic>10). It’s noteworthy that our study predominantly involved individuals of European descent, potentially limiting the generalizability of our study results to a more diverse population.

Furthermore, it’s essential to acknowledge that MR analysis is a hypothesis-based approach, and its outcomes can only establish associations rather than causal relationships. Subsequent experimental and clinical research is indispensable to establish the causal relationship between gut microbiota and specific diseases.

Additionally, there might be some subjectivity involved in eliminating the confounding effects of genetic variables through phenoscanner. This subjectivity could introduce some bias into our research results, emphasizing the importance of interpreting and understanding the findings with caution.

In conclusion, although our study has uncovered valuable insights, it’s imperative to recognize the aforementioned limitations. Future research should aim for more comprehensive and diversified investigations to further enhance our understanding of the intricate relationship between gut microbiota and diseases.

In summary, our study conducted a comprehensive assessment of the causal relationship between gut microbiota and Gastroduodenal ulcer. Our findings have contributed valuable insights and directions for further research on the prevention and treatment of Gastroduodenal ulcer. However, while we have acquired an initial understanding of the connection between gut microbiota and Gastroduodenal ulcer, the precise mechanisms underlying the role of gut microbiota in this condition remain unclear. Our study has established a correlation, but further research is necessary to elucidate how gut microbiota influences the occurrence and progression of Gastroduodenal ulcer.

In future investigations, we plan to delve deeper into the mechanisms through which gut microbiota contributes to the development of Gastroduodenal ulcer. This will involve analyzing the composition and functionality of the gut microbiota and its interactions with the host. Such endeavors will enhance our comprehension of the relationship between gut microbiota and Gastroduodenal ulcer, offering more targeted approaches for the prevention and treatment of this condition.

Moreover, while our study has uncovered pivotal insights into the relationship between gut microbiota and Gastroduodenal ulcer through Mendelian Randomization (MR), it’s essential to acknowledge the inherent limitations of this method. MR, by nature, establishes associations but doesn’t conclusively prove causation. It relies on genetic variance, lacking the capacity for direct exposure manipulation present in randomized controlled trials. Hence, it’s imperative to recognize the need for additional experimental and clinical research to validate and establish causative relationships between specific exposures and health outcomes, which can provide more direct and conclusive evidence.

## Data availability statement

The datasets presented in this study can be found in online repositories. The names of the repository/repositories and accession number(s) can be found in the article/**Supplementary Material**.

## Author contributions

YW: Conceptualization, Data curation, Formal analysis, Investigation, Methodology, Project administration, Software, Supervision, Writing – original draft, Writing – review & editing. JZ: Conceptualization, Data curation, Formal analysis, Investigation, Methodology, Project administration, Writing – original draft, Writing – review & editing. YH: Conceptualization, Data curation, Investigation, Methodology, Software, Supervision, Writing – original draft, Writing – review & editing. LW: Conceptualization, Data curation, Formal analysis, Funding acquisition, Investigation, Project administration, Software, Writing – original draft, Writing – review & editing. QZ: Conceptualization, Data curation, Formal analysis, Methodology, Supervision, Writing – original draft. BH: Formal Analysis, Project administration, Resources, Visualization, Writing – review & editing. ZL: Formal analysis, Funding acquisition, Project administration, Resources, Validation, Visualization, Writing – original draft.
