# Supplementary material for: Causal effect of gut microbiota on Gastroduodenal ulcer: a two-sample Mendelian randomization study
Source: Front Cell Infect Microbiol. 2023 Dec 8;13:1322537. doi: 10.3389/fcimb.2023.1322537 (PMC10753992; doi:10.3389/fcimb.2023.1322537)
Supplement: Supplementary file 1 [file DataSheet_1.pdf]

Supplementary Figures

Supplementary Figure S1 Forest plots of the causal effects of gut microbiota on the risk of Gastroduodenal ulcer.

(A)family Enterobacteriaceae; (B)family Streptococcaceae; (C)genus Butyricicoccus; (C) genus Candidatus Soleaferrea; (E) genus LachnospiraceaeNC2004group; (F) genus LachnospiraceaeUCG010; (G) genus Marvinbryantia; (H) genus Peptococcus; (I) genus Roseburia; (J) genus Streptococcus; (K)order Enterobacteriales; (L) order MollicutesRF9; (M) order NB1n.

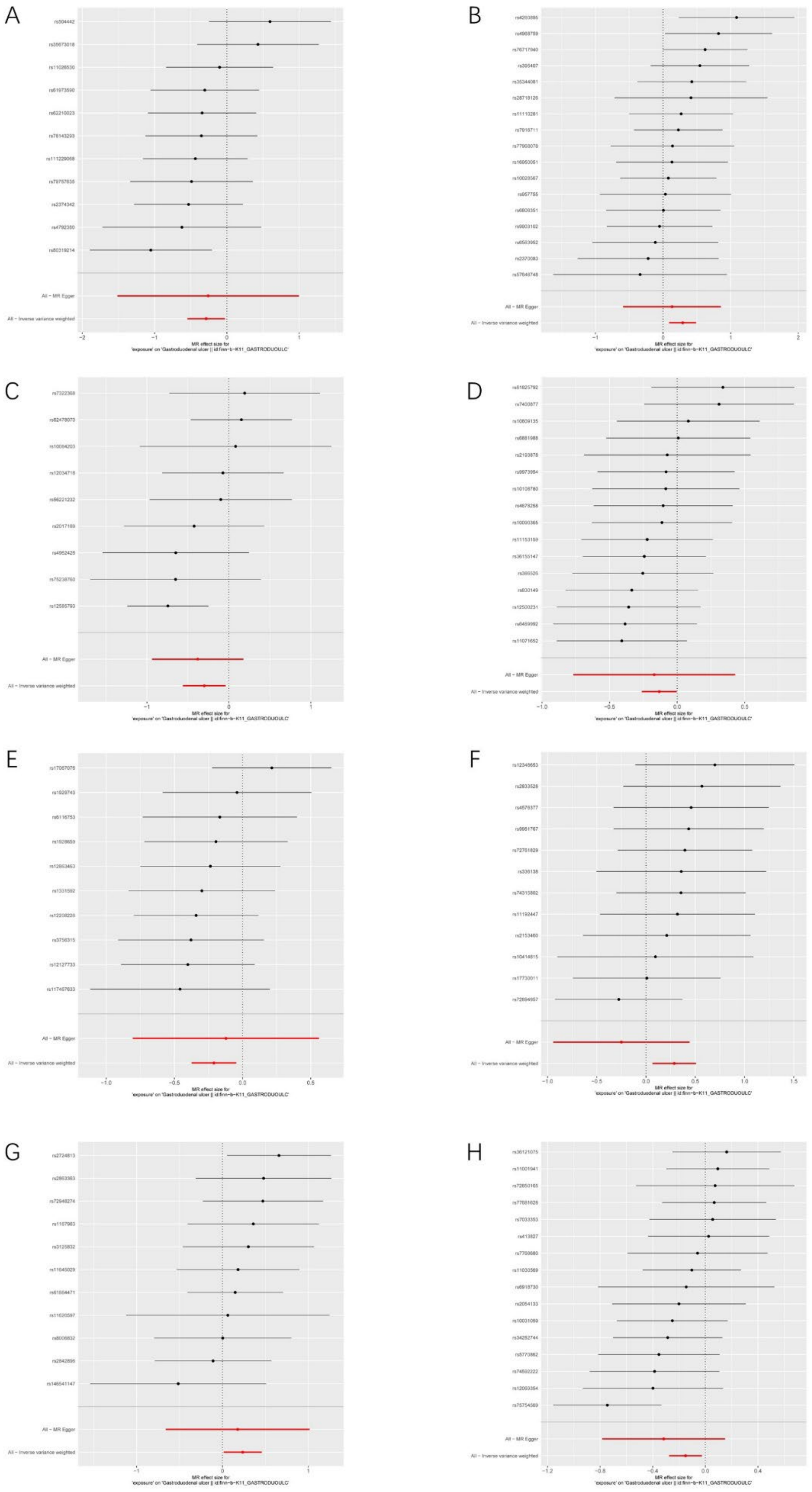
A
B
C
D
E
F
G
H
All - MR Egger
All - Inverse variance weighted
MR effect size for
'exposure' on 'Gastroduodenal ulcer || id:finn-b-K11_GASTRODUODULC'

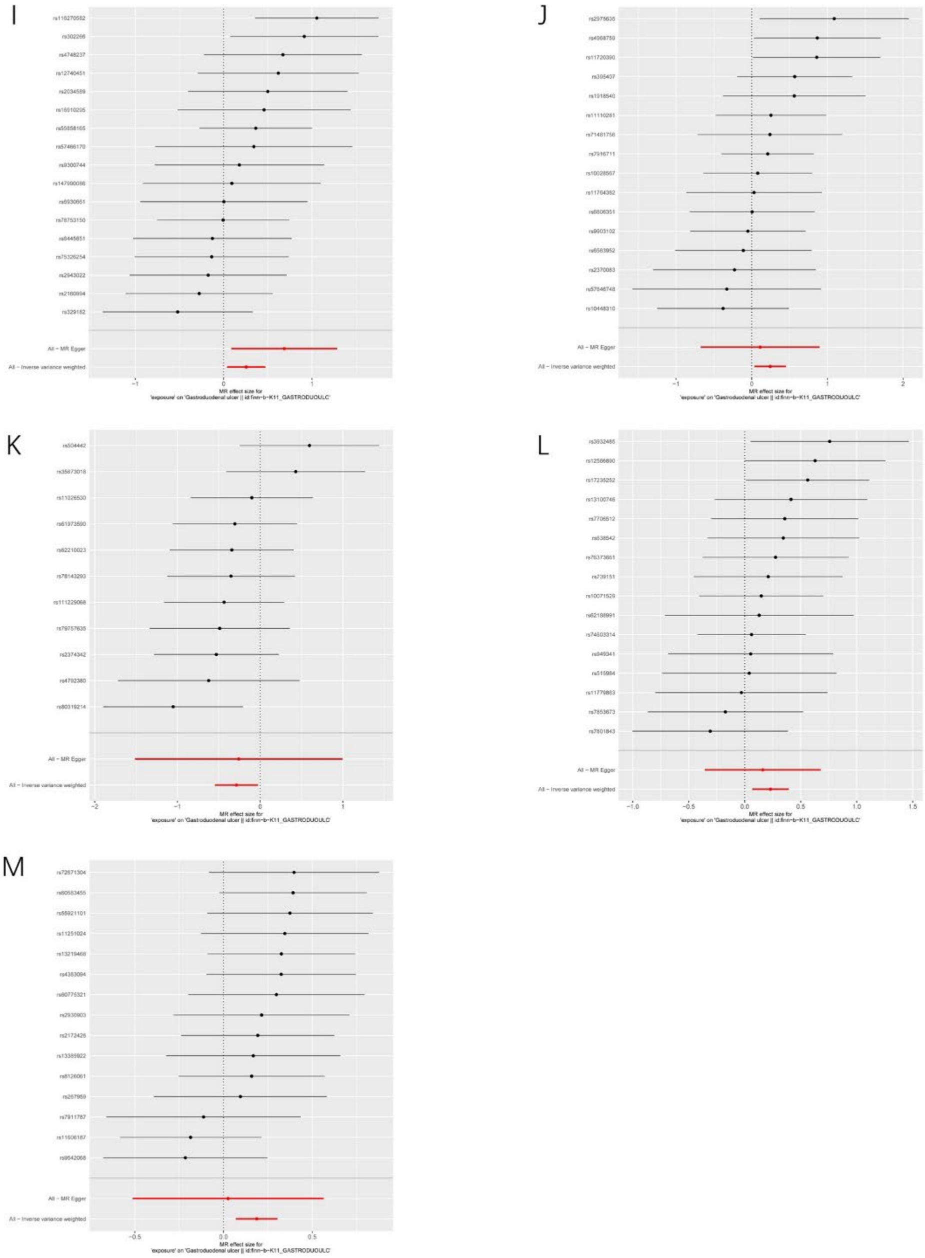

Supplementary Figure S2 Leave-one-out sensitivity analyses of the causal effects of gut microbiota on the risk of Gastroduodenal ulcer.

(A)family Enterobacteriaceae; (B)family Streptococcaceae; (C)genus Butyricicoccus; (C) genus Candidatus Soleaferrea; (E) genus LachnospiraceaeNC2004group; (F) genus LachnospiraceaeUCG010; (G) genus Marvinbryantia; (H) genus Peptococcus; (I) genus Roseburia; (J) genus Streptococcus; (K)order Enterobacteriales; (L) order MollicutesRF9; (M) order NB1n.

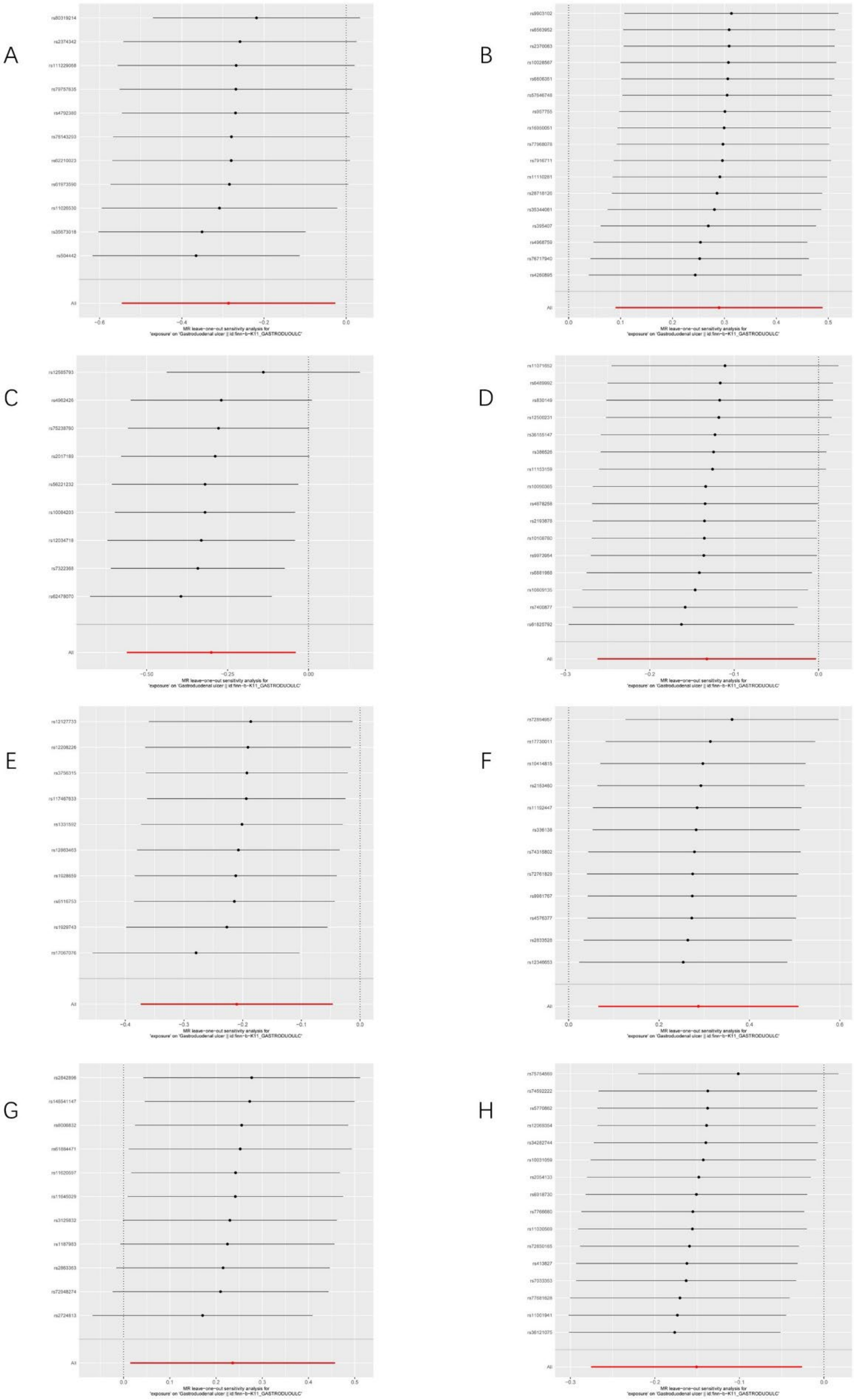
A
B
C
D
E
F
G
H
MR leave-one-out sensitivity analysis for
'exposure' on 'Gastroduodenal ulcer || id:finn-b-K11_GASTRODUOULC'

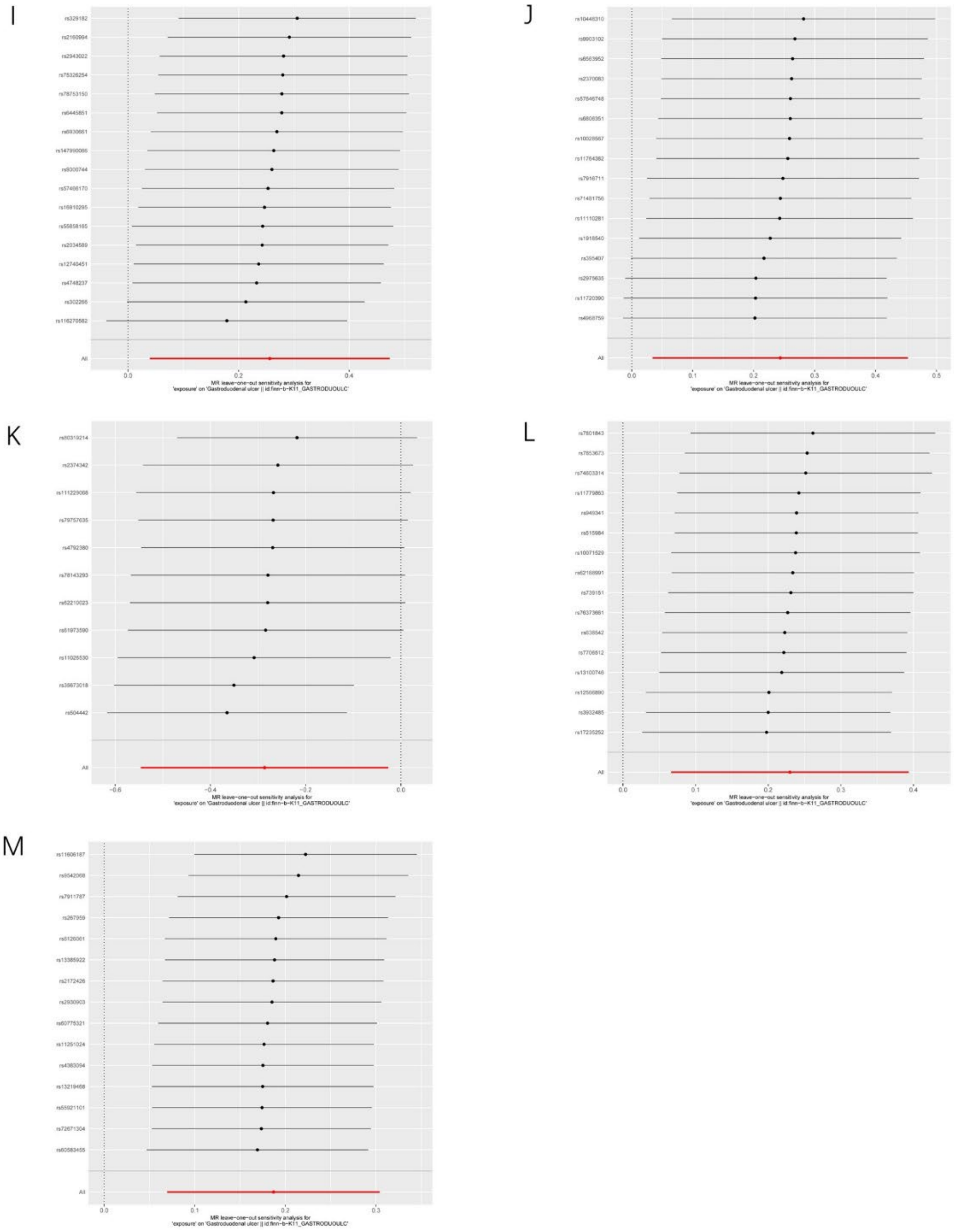

Supplementary Figure S3 Scatter plots of the causal effect of gut microbiota on the risk of Gastroduodenal ulcer.

(A)family Enterobacteriaceae; (B)family Streptococcaceae; (C)genus Butyricicoccus; (C) genus Candidatus Soleaferrea; (E) genus LachnospiraceaeNC2004group; (F) genus LachnospiraceaeUCG010; (G) genus Marvinbryantia; (H) genus Peptococcus; (I) genus Roseburia; (J) genus Streptococcus; (K)order Enterobacteriales; (L) order MollicutesRF9; (M) order NB1n.

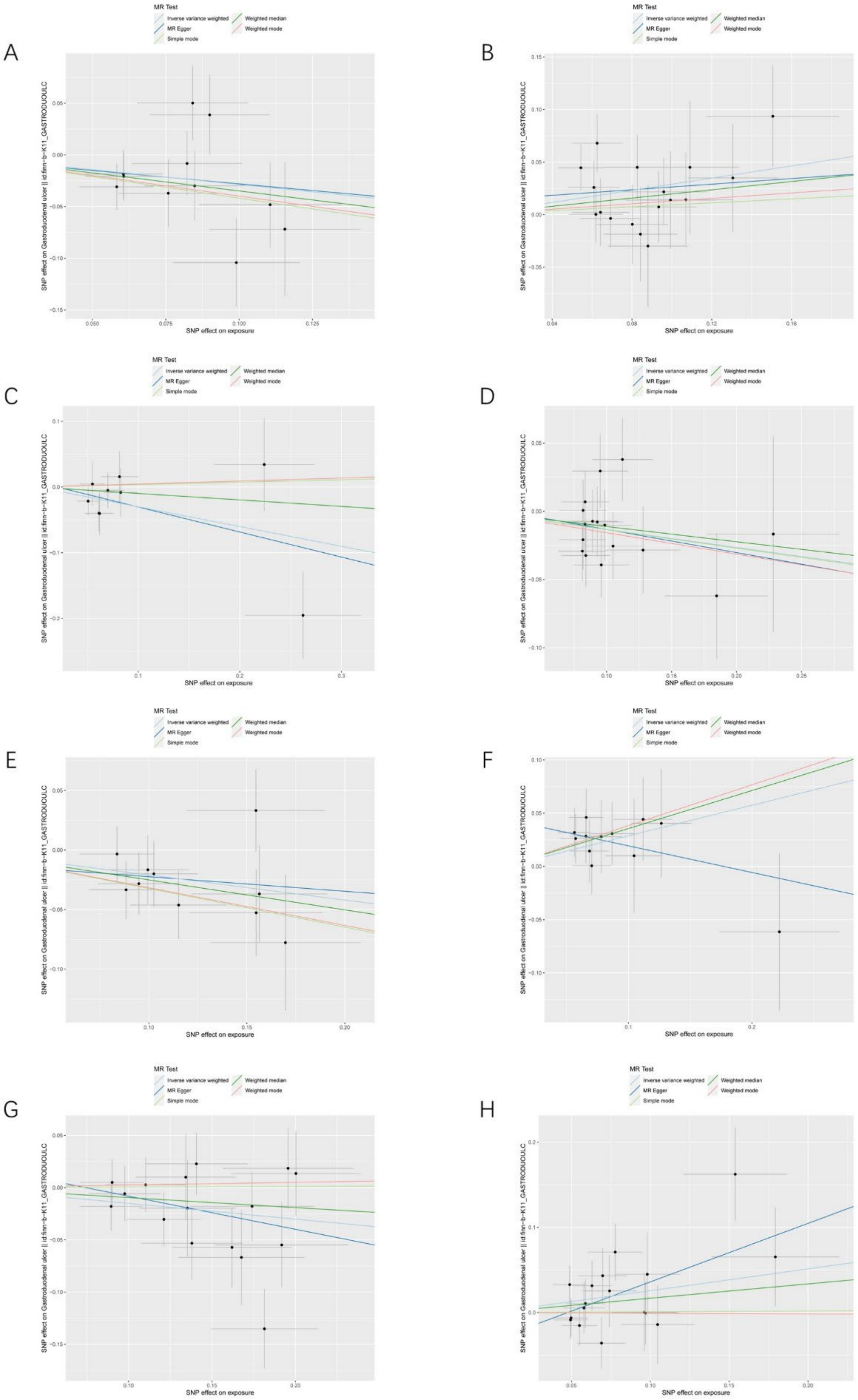
MR Test
Inverse variance weighted
MR Egger
Simple mode
Weighted median
Weighted mode
A
B
C
D
E
F
G
H
SNP effect on Gastroduodenal ulcer || id:finn-b-K11_GASTRODUOULC
SNP effect on exposure

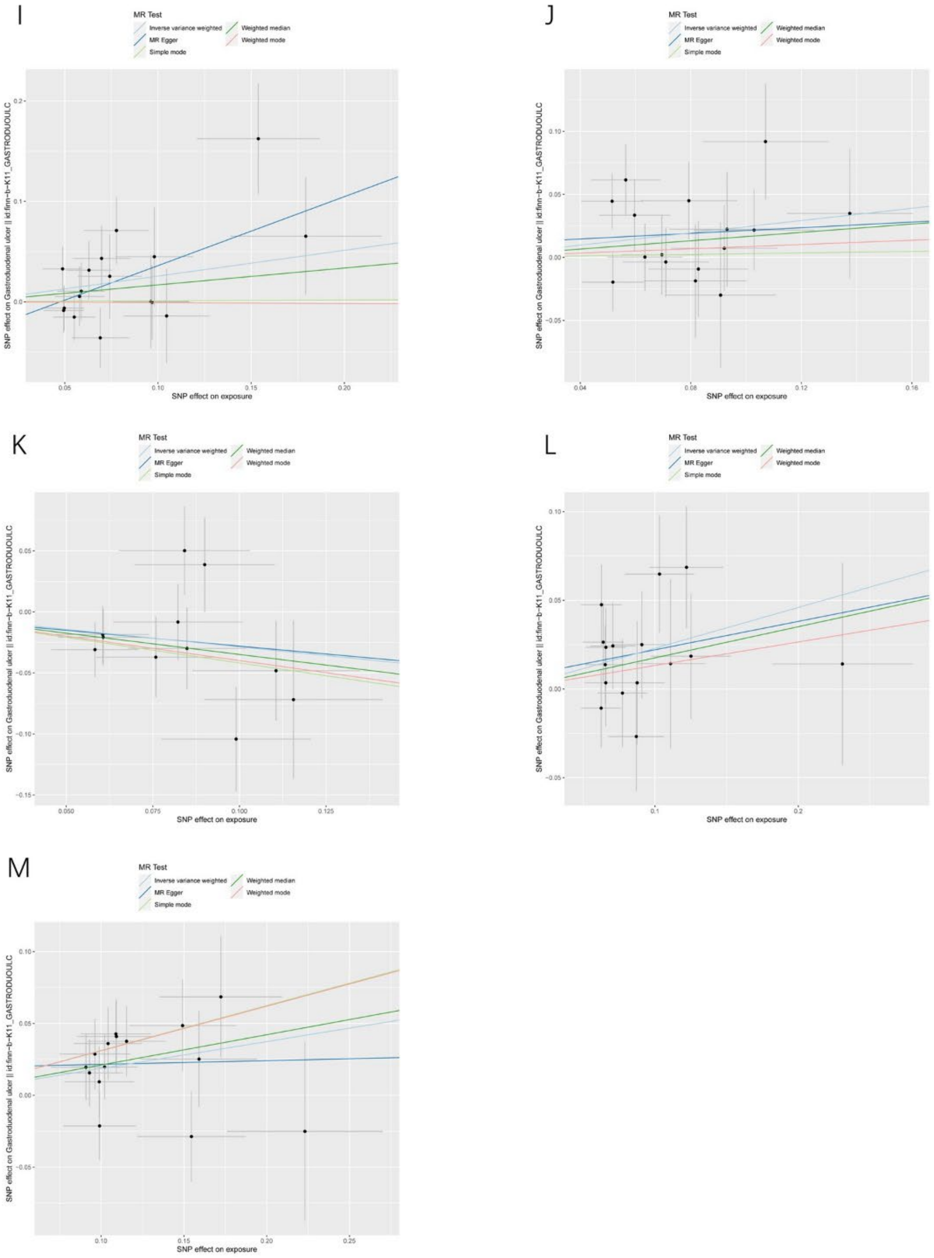

Supplementary Table S1.

SNP	beta.exposure	se.exposure	effect_allele.exposure	other_allele.exposure	pval.exposure	samplesize.exposure	R2	F
rs10028567	-0.0934	0.019	C	T	3.72E-06	16549	0.001446	24.07634
rs10031059	-0.12117	0.023	T	C	1.24E-07	5526	0.005182	28.77338
rs10071529	0.125105	0.028	G	C	8.64E-06	10147	0.004972	20.14361
rs10084203	0.05497	0.012	A	G	8.59E-06	17135	0.001154	19.78903
rs10090365	-0.08344	0.018	A	G	4.17E-06	6145	0.003447	21.24799

**rs10108780**	-0.09283	0.02	A	G	3.64E-06	5999	0.003501	21.5801
**rs10414815**	0.10453	0.023	T	C	4.24E-06	11564	0.001621	20.60636
**rs10448310**	-0.05179	0.011	A	G	3.31E-06	16384	0.00143	21.64305
**rs10809135**	0.083486	0.018	T	C	5.47E-06	5999	0.003397	20.93673
**rs11001941**	-0.19561	0.039	G	A	1.33E-06	5607	0.004481	24.86437
**rs11026530**	0.082241	0.019	T	C	9.43E-06	13485	0.001433	19.46777
**rs11030569**	-0.17402	0.037	A	T	3.13E-06	5329	0.0039	21.62663
**rs11071652**	-0.09588	0.021	C	G	7.83E-06	6144	0.00325	20.02991
**rs11110281**	-0.13055	0.023	T	C	1.40E-08	15316	0.002004	33.38342
**rs111229068**	0.110561	0.024	A	T	3.65E-06	12350	0.001539	20.91789
**rs11153159**	-0.12805	0.029	G	C	4.42E-06	6101	0.003264	20.11729
**rs11192447**	0.126592	0.024	A	G	4.69E-07	12695	0.002125	27.03494
**rs11251024**	0.104201	0.021	G	A	6.63E-07	5107	0.005171	25.39394
**rs11606187**	-0.15454	0.033	A	G	3.31E-06	5102	0.004569	22.42319
**rs11620597**	0.119479	0.027	T	C	7.80E-06	11031	0.00167	19.33617
**rs116270582**	-0.15384	0.033	T	A	1.20E-06	9099	0.001249	21.82047
**rs11645029**	-0.06062	0.013	G	C	4.15E-06	11709	0.001834	21.22945
**rs11720390**	0.107024	0.023	G	A	3.59E-06	15220	0.001454	22.00756
**rs117467633**	-0.16969	0.038	T	C	9.13E-06	5077	0.003478	19.60493
**rs11764382**	-0.06953	0.014	A	G	1.29E-06	16387	0.001547	23.42085
**rs11779863**	-0.07728	0.017	G	A	6.69E-06	10353	0.00497	20.13563
**rs1187983**	-0.09355	0.019	C	T	2.02E-06	11896	0.002025	23.44618

**rs12034718**	0.07012	0.016	A	G	9.58E-06	16237	0.001145	19.64015
**rs12069354**	0.16763	0.038	C	T	9.28E-06	5352	0.003518	19.50386
**rs12127733**	0.115182	0.025	G	A	3.11E-06	6226	0.003885	21.91386
**rs12208226**	-0.15474	0.034	C	A	9.75E-06	5620	0.003664	20.66033
**rs12346653**	0.065769	0.014	C	T	2.70E-06	12816	0.001746	22.20182
**rs12500231**	0.081385	0.018	A	T	7.68E-06	6145	0.003219	19.83787
**rs12566890**	-0.10313	0.024	T	G	8.11E-06	10339	0.004492	18.18709
**rs12585793**	-0.26221	0.056	T	C	5.79E-06	3135	0.001257	21.55537
**rs12740451**	0.069753	0.015	T	C	7.34E-06	17837	0.001181	20.6181
**rs12863463**	-0.15636	0.035	G	A	6.04E-06	5516	0.003634	20.49042
**rs13100746**	0.063898	0.014	C	T	7.29E-06	10359	0.004948	20.04465
**rs13219468**	0.115344	0.024	G	C	1.41E-06	5104	0.004825	23.68467
**rs1331592**	0.094881	0.021	C	G	5.34E-06	6328	0.003676	20.72694
**rs13385922**	0.092949	0.02	T	C	3.97E-06	5106	0.004342	21.30198
**rs146541147**	0.118845	0.027	G	A	6.86E-06	10778	0.001693	19.59972
**rs147990086**	-0.05789	0.013	A	G	8.93E-06	17840	0.001095	19.11771
**rs16910295**	-0.09804	0.021	T	C	2.91E-06	16648	0.001253	21.88415
**rs16950051**	0.107008	0.024	A	G	5.34E-06	14431	0.001225	20.38821
**rs17067076**	-0.15463	0.035	G	A	5.61E-06	4449	0.003418	19.2698
**rs17235252**	-0.122	0.026	T	C	2.16E-06	9531	0.005623	22.79626
**rs17730011**	-0.07024	0.016	G	A	7.85E-06	13226	0.001573	19.99472
**rs1918540**	0.059639	0.013	G	A	2.44E-06	16373	0.00143	21.65606

**rs1928659**	0.102522	0.023	T	C	6.17E-06	5516	0.003634	20.49051
**rs1929743**	0.083721	0.019	T	C	9.06E-06	6330	0.003431	19.3441
**rs2017189**	-0.0507	0.011	G	T	3.87E-06	17133	0.001233	21.1451
**rs2034589**	0.062976	0.012	G	C	5.01E-07	17846	0.001489	26.0046
**rs2054133**	0.089543	0.019	G	A	2.14E-06	5657	0.004074	22.59761
**rs2153460**	-0.0684	0.016	A	T	9.17E-06	13236	0.001501	19.08628
**rs2160994**	0.055069	0.011	T	C	9.70E-07	17444	0.001372	23.96627
**rs2172426**	-0.10212	0.02	C	T	3.17E-07	5099	0.005363	26.33719
**rs2193878**	0.228255	0.051	T	A	9.46E-06	3088	0.003259	20.0841
**rs2370083**	-0.08428	0.018	G	T	4.26E-06	16516	0.001253	20.85976
**rs2374342**	0.058293	0.013	C	A	4.52E-06	13577	0.00157	21.33448
**rs267959**	0.098861	0.021	A	G	2.62E-06	5107	0.004528	22.22175
**rs2724813**	-0.08408	0.017	A	G	6.28E-07	11610	0.002174	25.17593
**rs2833528**	-0.05621	0.013	C	T	9.92E-06	13233	0.001523	19.35633
**rs2842896**	-0.06494	0.013	C	T	7.25E-07	11709	0.002117	24.51494
**rs2863363**	0.063486	0.014	A	G	3.11E-06	11950	0.001873	21.68426
**rs28718126**	0.109069	0.025	A	G	9.41E-06	13529	0.001173	19.51742
**rs2930903**	0.090833	0.021	C	G	6.84E-06	4789	0.003834	18.80273
**rs2943022**	0.049379	0.011	T	C	4.11E-06	17854	0.001225	21.38861
**rs2975635**	-0.05645	0.013	T	C	7.04E-06	16382	0.001336	20.22428
**rs302266**	-0.07773	0.017	T	C	8.13E-06	17056	0.001156	20.18936
**rs3125832**	0.067932	0.015	A	C	5.03E-06	11709	0.001768	20.4739

**rs329182**	0.069033	0.015	T	C	5.90E-06	17854	0.001167	20.38617
**rs336138**	0.077954	0.017	G	T	7.48E-06	13236	0.001618	20.57001
**rs34282744**	0.191797	0.04	G	C	1.84E-06	5257	0.004147	23.00235
**rs35344081**	0.060935	0.013	G	A	2.64E-06	16624	0.001326	22.06876
**rs35673018**	0.089964	0.02	G	A	7.63E-06	13326	0.001446	19.65008
**rs36121075**	-0.14067	0.031	A	G	6.99E-06	5301	0.003803	21.08673
**rs36155147**	0.104966	0.024	C	T	5.41E-06	5958	0.003078	18.96444
**rs3756315**	-0.08835	0.019	A	G	3.33E-06	6328	0.003898	21.98272
**rs386526**	0.081846	0.018	C	G	8.33E-06	6144	0.00335	20.64863
**rs3932485**	0.062621	0.014	C	T	9.93E-06	10359	0.004824	19.54133
**rs395407**	-0.08269	0.017	G	C	1.33E-06	16088	0.00138	22.96395
**rs413827**	0.110229	0.024	G	A	3.30E-06	5482	0.003882	21.52906
**rs4260895**	-0.06256	0.013	C	A	4.50E-07	16627	0.001497	24.92426
**rs4383094**	0.149188	0.032	T	C	4.28E-06	5091	0.00441	21.63689
**rs4576377**	-0.0572	0.013	A	C	7.63E-06	13225	0.001594	20.2686
**rs4678258**	0.098614	0.022	T	C	5.53E-06	6101	0.003392	20.90492
**rs4748237**	0.048833	0.011	G	C	4.67E-06	17851	0.001206	21.05968
**rs4792380**	0.115598	0.026	A	T	9.49E-06	12763	0.001481	20.12167
**rs4962426**	0.061422	0.014	G	T	7.38E-06	16889	0.001189	20.40078
**rs4968759**	-0.0544	0.011	A	G	8.92E-07	16624	0.001436	23.90207
**rs504442**	0.084159	0.019	T	G	5.17E-06	13578	0.001451	19.72494
**rs515984**	-0.08755	0.019	T	C	6.61E-06	10359	0.005199	21.06756

**rs55858165**	0.179284	0.04	A	C	9.99E-06	5447	0.001122	19.59853
**rs55921101**	-0.10925	0.024	A	T	4.32E-06	5105	0.004358	21.38289
**rs56221232**	0.082803	0.017	T	C	7.62E-07	17133	0.001426	24.46382
**rs57466170**	0.074141	0.017	C	T	8.30E-06	17333	0.001069	18.66474
**rs57646748**	-0.08802	0.02	G	A	7.88E-06	16160	0.001178	19.60939
**rs5770862**	0.162018	0.036	T	C	3.22E-06	5431	0.003717	20.61039
**rs60583455**	0.108934	0.021	T	C	2.60E-07	4773	0.005385	26.44958
**rs60775321**	-0.09624	0.021	T	C	7.10E-06	5101	0.004128	20.2475
**rs6116753**	0.099475	0.021	G	A	2.92E-06	6278	0.004009	22.6152
**rs61825792**	0.112199	0.023	T	C	1.37E-06	6145	0.003796	23.41001
**rs61884471**	0.124426	0.025	G	A	1.01E-06	11559	0.002165	25.08056
**rs61973590**	-0.06054	0.013	C	G	8.54E-06	13570	0.001508	20.50083
**rs62188991**	-0.11086	0.024	G	C	5.27E-06	9531	0.005204	21.08885
**rs62210023**	0.060675	0.013	A	G	3.13E-06	13515	0.001599	21.73899
**rs62478070**	0.224039	0.049	T	G	5.94E-06	4007	0.001194	20.48608
**rs638542**	-0.07059	0.016	G	A	5.17E-06	9970	0.004985	20.19412
**rs6445851**	-0.04973	0.011	G	A	3.53E-06	17851	0.001211	21.14124
**rs6489992**	-0.08404	0.019	A	G	7.89E-06	6142	0.003275	20.18569
**rs6563952**	0.080193	0.018	G	C	8.71E-06	16627	0.001212	20.16395
**rs6806351**	-0.06192	0.014	T	C	6.94E-06	16198	0.00125	20.80566
**rs6881988**	-0.08191	0.018	G	C	9.23E-06	6140	0.003291	20.28323
**rs6918730**	0.135311	0.029	G	A	1.15E-06	5656	0.003931	21.80135

**rs6930661**	-0.09616	0.02	C	T	2.48E-06	16945	0.00126	22.00528
**rs7033353**	0.090152	0.019	T	G	2.22E-06	5657	0.00406	22.51721
**rs71481756**	0.093105	0.021	T	G	6.51E-06	14526	0.001324	20.04343
**rs72671304**	0.172322	0.037	T	C	3.80E-06	4887	0.004418	21.6787
**rs72761829**	0.111884	0.024	A	T	2.58E-06	11488	0.001725	21.93098
**rs72850165**	-0.1343	0.03	T	C	5.74E-06	5627	0.003604	19.97809
**rs72894957**	0.222263	0.049	G	A	5.68E-06	4316	0.001642	20.87535
**rs72948274**	-0.12635	0.027	A	C	3.26E-06	11561	0.001861	21.54214
**rs7322368**	0.081573	0.018	T	C	5.52E-06	17134	0.001156	19.8314
**rs739151**	0.065266	0.014	C	G	3.09E-06	10337	0.005377	21.79273
**rs7400877**	-0.09511	0.021	T	C	9.29E-06	6145	0.00324	19.96906
**rs74315802**	0.086726	0.018	G	T	3.19E-06	12715	0.001757	22.33925
**rs74592222**	0.137957	0.03	G	A	8.55E-06	5329	0.003738	20.72744
**rs74603314**	0.230767	0.049	T	C	2.28E-06	4033	0.005476	22.19578
**rs75238760**	0.061942	0.014	T	A	6.80E-06	17022	0.001142	19.58974
**rs75326254**	-0.10463	0.023	C	T	7.50E-06	15893	0.001176	20.53089
**rs75754569**	0.181434	0.032	C	G	1.10E-08	5481	0.005804	32.25023
**rs76373661**	0.090822	0.02	G	A	5.16E-06	10359	0.004939	20.00934
**rs76717940**	0.150606	0.033	T	A	3.09E-06	7720	0.001221	20.32035
**rs7706512**	0.065735	0.014	G	A	2.27E-06	10359	0.005517	22.36038
**rs7766680**	0.097683	0.021	G	C	3.51E-06	5655	0.003751	20.79822
**rs77681628**	0.200307	0.039	C	T	2.69E-07	4842	0.004816	26.73494

**rs77968078**	-0.0993	0.022	G	A	7.93E-06	15519	0.001173	19.5125
**rs7801843**	-0.08695	0.019	A	G	9.47E-06	10359	0.00493	19.97272
**rs78143293**	-0.08485	0.017	A	G	1.20E-06	13572	0.001823	24.78819
**rs7853673**	-0.06244	0.014	G	A	6.73E-06	10359	0.00492	19.92872
**rs78753150**	0.096874	0.021	A	C	9.98E-06	16303	0.001173	20.47693
**rs7911787**	-0.22307	0.047	G	T	3.39E-06	3879	0.004584	22.49477
**rs7916711**	0.095964	0.022	A	G	6.33E-06	15515	0.001192	19.8367
**rs79757635**	0.07586	0.017	C	A	9.32E-06	13419	0.001443	19.61174
**rs8006832**	-0.09524	0.022	G	T	6.58E-06	11412	0.001668	19.31369
**rs80319214**	0.09904	0.022	C	G	6.95E-06	12641	0.001548	21.04487
**rs8126061**	-0.15914	0.035	T	C	7.36E-06	5089	0.004167	20.44118
**rs830149**	0.184647	0.04	C	G	9.58E-06	5065	0.003504	21.59936
**rs9300744**	-0.05885	0.013	C	T	4.75E-06	17854	0.001244	21.73008
**rs949341**	0.06566	0.015	G	A	7.73E-06	10355	0.004908	19.88276
**rs9542068**	0.099071	0.022	T	C	6.52E-06	5107	0.004203	20.61611
**rs957755**	-0.06424	0.014	T	G	7.42E-06	16627	0.001218	20.26575
**rs9903102**	-0.0693	0.015	C	A	4.92E-06	16623	0.001215	20.22316
**rs9973954**	0.089208	0.02	A	G	5.95E-06	6145	0.00338	20.83559
**rs9981767**	0.065506	0.013	A	C	9.96E-07	12816	0.001936	24.62561
